# Reliable gains? Evidence for substantially underpowered designs in studies of working memory training transfer to fluid intelligence

**DOI:** 10.3389/fpsyg.2014.01589

**Published:** 2015-01-22

**Authors:** Tim Bogg, Leanne Lasecki

**Affiliations:** Department of Psychology, Wayne State UniversityDetroit, MI, USA

**Keywords:** cognitive training intervention, transfer effects, fluid intelligence, meta-analysis, statistical power, small-study effects

## Abstract

In recent years, cognitive scientists and commercial interests (e.g., Fit Brains, Lumosity) have focused research attention and financial resources on cognitive tasks, especially working memory tasks, to explore and exploit possible transfer effects to general cognitive abilities, such as fluid intelligence. The increased research attention has produced mixed findings, as well as contention about the disposition of the evidence base. To address this contention, Au et al. (2014) recently conducted a meta-analysis of extant controlled experimental studies of *n*-back task training transfer effects on measures of fluid intelligence in healthy adults; the results of which showed a small training transfer effect. Using several approaches, the current review evaluated and re-analyzed the meta-analytic data for the presence of two different forms of small-study effects: (1) publication bias in the presence of low power and; (2) low power in the absence of publication bias. The results of these approaches showed no evidence of selection bias in the working memory training literature, but did show evidence of small-study effects related to low power in the absence of publication bias. While the effect size estimate identified by Au et al. (2014) provided the most precise estimate to date, it should be interpreted in the context of a uniformly low-powered base of evidence. The present work concludes with a brief set of considerations for assessing the adequacy of a body of research findings for the application of meta-analytic techniques.

## INTRODUCTION

The pursuit of evidence suggesting the malleability of cognitive abilities has long been an interest in cognitive science, and psychological science, more generally. One of the recent forms of this pursuit has been studies examining working memory training transfer effects to fluid intelligence (e.g., Jaeggi et al., 2008; Morrison and Chein, 2011; Thompson et al., 2013). Due to its role in short-term memory capacity, resistance to distraction, mental manipulation, attentional control, and maintenance of memory traces (Baddeley and Logie, 1999; Cowan, 1999; Unsworth and Engle, 2007), working memory capacity is posited to act as an important lower-order substrate of the higher-order cognitive abilities of abstract reasoning and problem solving (i.e., fluid intelligence). Fluid intelligence, in turn, is associated with performance across a variety of consequential life domains (Nisbett et al., 2012). To the extent working memory is closely linked with fluid intelligence, then the hypothesized mechanism for improving fluid intelligence is via training gains and concomitant transfer effects from working memory tasks. Indeed, the studies collected in a recent meta-analysis of working memory transfer effects to fluid intelligence were screened for inclusion based on this design framework, using a control condition for comparison with the training condition (Au et al., 2014).

The meta-analytic review conducted by Au et al. (2014) focused on studies using adaptive single or dual *n*-back working memory tasks for training sessions with healthy adults (where inclusion criteria also required training sessions to last for more than 1 week; individual study session lengths were 18.5 min and longer). In an adaptive single *n*-back task, individuals monitor audible or visible stimuli (e.g., a letter, the location of a shape on a computer screen) over a series of trials and indicate whether the stimulus appearing in the current trial is the same as or different from that of *n* trials back. The adaptive component of these tasks uses a post-block performance algorithm to increase or decrease *n* for the next block of trials. Twenty-two unique measures of fluid intelligence were used across the 24 independent samples identified by Au et al. (2014) including the BOMAT, WAIS, and Raven’s Advanced Progressive Matrices [a complete list of fluid intelligence measures appears in supplemental Table 3 of Au et al. (2014)]. Using random effects analyses and conversion of standardized mean differences to Hedge’s *g*s, the overall effect size across the 24 samples was *g* = 0.24 (SE = 0.069), indicating a small transfer effect of working memory training on fluid intelligence. Although the test statistic for examining heterogeneity (*I*^2^) was quite small (6.92%) and non-significant, additional moderation and meta-regression analyses were conducted as well.

In spite of what appears to be a straightforward meta-analytic finding derived from an emergent literature, concerns regarding the reliability of the effect are raised by the small reported average sizes of the control and treatment groups across the studies (*M* = 19.29, SD = 8.74; and *M* = 19.96, SD = 8.13, respectively), as well as the dearth of confidence intervals (4 out of 24) that excluded zero in the reported forest plot [see Au et al. (2014) Figure 3]. These features of the included studies are highly suggestive of an under-powered base of literature – one that might hinder attempts to meta-analytically refine a provisional effect size estimate of the working memory training transfer effect. The present review provides an evaluation and re-analysis of the meta-analytic database in an attempt to account and control for small-study effects, a problem found to be alarmingly prevalent across received psychological findings (e.g., the ego depletion effect; Carter and McCullough, 2014), as well as scientific disciplines more generally, including neuroscience and biomedical research (Ioannidis, 2005; Button et al., 2013). Specifically, small-study effects are defined as the low reliability of research findings that is attributable to (1) *biased* selection practices (e.g., publication bias) in small-sample designs that favor statistically significant results and (2) *unbiased* design practices and choices that result in very low-powered studies that produce both spurious significant and non-significant effects.

In the current review, two forms of small-study effects will be examined (cf. Button et al., 2013): (1) publication bias in the presence of low power; and (2) low power in the absence of publication bias. Small-study effects related to publication bias in the presence of low power are characterized by an overrepresentation of large and statistically significant effects compared to smaller non-significant effects. This overrepresentation of large and significant effects can result from bias in the selection of data analyses (e.g., idiosyncratic and/or significance-serving data inclusion/exclusion procedures) and/or bias in the selection of outcomes that are reported (e.g., only communicating findings for measures that showed significant effects). Small-study effects related to low power in the absence of publication bias are characterized by an under-powered base of literature for which there is no evidence of a systematic pattern of selection bias, but for which heavily inflated Type II error rates (likelihood of rejecting a truly non-null effect) result in a limited number of possibly spurious, but large effects, and a greater number of non-zero null effects that are statistically indistinguishable from zero due to sampling error and random error. In the current work, several approaches are used to test for the absence or presence of these two forms of small-study effects.

## MATERIALS AND METHODS

Three sets of approaches were used to examine small-study effects related to publication bias in the presence of low power using the data reported by Au et al. (2014; their Figure 3; a forest plot, provides the Hedge’s *g* effect size estimate and corresponding SE for each sample). First, a contour-enhanced funnel plot was created to visualize the distribution of effects that are statistically non-significant versus significant. A disproportionate number of studies missing from the region of non-significance serves as an indication of publication bias (Peters et al., 2008). Second, a binomial test for a surplus of significant findings was conducted (Ioannidis and Trikalinos, 2007). This test examines whether the number of significant effects in a given set of studies is greater than the number of significant effects that should be expected given the mean power of the studies. In the binomial test, smaller *p* values indicate a scarcity of null findings and a surplus of significant findings. Third, an elaboration of Egger’s regression test was used as an additional test of the relationship between effect size and SE (explained in detail below; Stanley and Doucouliagos, 2007, 2013).

To test for the possibility of small-study effects related to low power in the absence of publication bias, three approaches were used. First, a *post hoc* power estimate was calculated to examine the average statistical power available in the 24 independent samples to detect Hedge’s *g* = 0.24. This analysis used the mean treatment (*n* = 19.96) and control (*n* = 19.29) cell sizes reported by Au et al. (2014). For illustrative purposes, an additional power analysis was conducted using the upper end of the range for treatment (*n* = 36) and control (*n* = 43) groups (i.e., assuming the mean group size was at the upper end of reported group sizes). *Post hoc* power analyses of the primary studies from an extant meta-analysis can be instructive with regard to the design of future primary studies. A related set of retrospective and prospective meta-analytic power analyses was conducted to test how power varied as a function of assumptions related to effect size, sample sizes, amount of heterogeneity, and the number of studies in the meta-analytic database (Hedges and Pigott, 2001). Valentine et al. (2010) advised that retrospective meta-analytic power analyses are not informative when they are based on the observed estimates of the modeling procedure (e.g., values from a random-effects model). Such power analyses are circular in that they use values produced from a test that, by definition, already rendered significant or non-significant results. To avoid such circularity, the retrospective and prospective power analyses were conducted using varying values of Hedge’s *g*, group sample sizes, amounts of heterogeneity, and number of studies. Consistent with the suggestions of Hedges and Pigott (2001) and Valentine et al. (2010), we selected one conservative estimate for the overall effect size from the lower bound of the observed confidence interval from the random-effects model (i.e., Hedge’s *g* = 0.11). We also used the observed estimate (Hedge’s *g* = 0.24) and the upper bound of the observed confidence interval (Hedge’s *g* = 0.38). The retrospective power analyses were conducted using a near approximation of the average group sizes (rounded to *n* = 20), with small amounts of heterogeneity (where τ^2^ = 0.33*v* via convention for low heterogeneity, where *v* is the estimate of the common variance of effect sizes across studies; Hedges and Pigott, 2001) as was observed in the random-effects model. For the prospective power analyses, *k* was increased by 50% (i.e., *k* = 36), group sizes were varied in a twofold manner (*ns*_treatment,control_ = 20 versus 40), and the amounts of heterogeneity were varied (small versus large, τ^2^ = 0.33*v* and τ^2^ = 1*v*; Hedges and Pigott, 2001). All meta-analytic power analyses were conducted using equations provided by Hedges and Pigott (2001).

Second, two sets of positive predictive value (PPV) analyses were conducted to examine (1) the probability that at least one study effect among several study effects on the same research question would reflect a true effect, and (2) the probability that an observed meta-analytic effect would reflect a true effect (i.e., a true positive result). For the probability that at least one study effect among several study effects on the same research question reflects a true finding, PPV and statistical power are positively correlated, where PPV = R(1 - β)/(R + 1 -[1 - α]^n^ - Rβ^n^) (Ioannidis, 2005). In this equation, (1 - β) is statistical power (where β is the Type II or false-negative error rate, i.e., erroneously rejecting a truly non-null effect), *R* is the odds that an effect is truly non-null based on an *a priori* judgment, α is the Type I (false positive) error rate (e.g., *p* < 0.05), and *n* is the number of studies with similar statistical power performed on a given research question (for this form of PPV analysis, *n* is interchangeable with the meta-analytic notation, *k*). It should be noted that the PPV calculation used here does not correct for estimates of bias (represented by the term *u* in other calculations of PPV) and therefore can indicate small-study effects related to low power in the absence of publication bias. Using a range of pre-study odds (i.e., 1 in 8, 1 in 4, and 1 in 2), the meta-analytic PPV analyses used the varying assumptions described above for the retrospective and prospective meta-analytic power analyses.

The third approach used to test for possible small-study effects related to publication bias in the presence of low power *and* small-study effects related to low power in the absence of publication bias was a two-part conditional extension of Egger’s regression test – precision-effect test/precision effect estimate with SE (PET-PEESE; Stanley and Doucouliagos, 2007, 2013). PET-PEESE can offer a more precise account of the impact of small-study effects on the effect size estimate. In Egger’s regression test (Egger and Sterne, 2005), funnel plot asymmetry (plotting effect sizes by their corresponding SE) is evaluated formally using a weighted least squares (WLS) regression model where the effect size is predicted by the SE. A statistically significant slope coefficient in this regression (i.e., the predictive effect of the SE) indicates funnel plot asymmetry.

The PET furthers the use of Egger’s regression to the interpretation of the intercept coefficient as an estimate of the effect size when the SE equals zero (hence, the use of “precision” to suggest a completely precise study; Stanley, 2005). A conditional adjunct to PET is the PEESE. In simulation research, Stanley and Doucouliagos (2007) found that PET was overly conservative when the true effect was non-zero. To compensate for this, the variance could be used instead of the SE in the WLS regression model. Stanley and Doucouliagos (2013) recommended the following conditional decision framework regarding PET-PEESE: (1) If the intercept coefficient for PET is statistically significant (i.e., non-zero), then the intercept coefficient from PEESE will provide the estimate of the overall effect; (2) If the intercept coefficient for PET is not statistically significant (i.e., indistinguishable from zero), then the intercept coefficient for PET should be retained as the estimate of the overall effect.

Support for the conditional PET-PEESE approach is borne out in simulation studies and re-analyses of extant meta-analytic databases (Stanley, 2008; Turner et al., 2008; Moreno et al., 2009; Carter and McCullough, 2014). Meta-analytic and simulation research indicates these approaches can reproduce an effect size of similar magnitude as conventional approaches under conditions absent of small-study effects (Moreno et al., 2009; Stanley and Doucouliagos, 2013). A strong and clever demonstration of this compared trials of antidepressant drugs registered with the U.S. Food and Drug Administration to peer-reviewed published research using data from the same registered trials (Turner et al., 2008). In this design, the analysis of data from the FDA registry represented a bias-free estimate of efficacy, owing to the absence of selection effects for inclusion in the registry. The results showed the PET-PEESE adjustment produced an effect size for the peer-reviewed published estimate that was nearly identical to that of the putatively unbiased data of the FDA registry. It should be noted that the rationale for regression-based approaches is not specifically tied to publication bias (as is the case with trim and fill analyses), but is broadly applicable to small-study effects, whatever the source of those effects might be (Rücker et al., 2011).

Annotated R scripts for all reported analyses are included in the Supplementary materials.

## RESULTS

**FIGURE 1:**
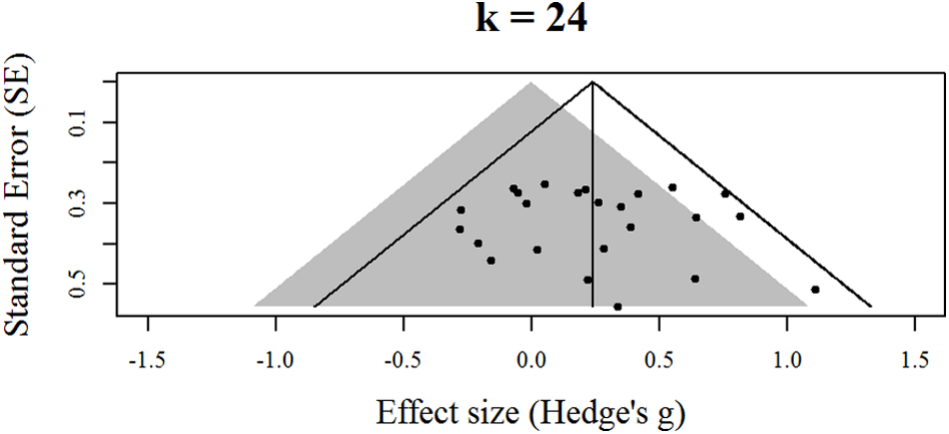
**Contour-enhanced funnel plot for effects identified by Au et al. (2014)**.

**Figure 1** displays the contour-enhanced funnel plot for the 24 effects identified by Au et al. (2014). The gray area represents statistical non-significance. The solid vertical line is the random-effects estimate of the overall effect size. The solid angled lines are the boundaries where 95% of the effects should reside in the absence of statistical heterogeneity. As can be seen, the funnel plot shows the vast majority of effects (20) are located in the area of non-significance and that all the effects fall within or upon the boundaries for statistical heterogeneity, which is indicative of low statistical heterogeneity. Results from the binomial test for a surplus of significant findings produced a moderate-sized *p* value (*p* = 0.29). Finally, **Table 1** displays the two slope terms for the PET-PEESE analyses. Both sets of confidence intervals for the slope terms included zero, indicating the absence of a meaningful association between effect size and SE. Taken together, the contour-enhanced funnel plot, the binomial test, and the slope terms for the PET-PEESE analyses are not suggestive of small-study effects related to publication bias in the presence of low power.

**Table 1:** Results from fixed effects, random effects, PET, and PEESE models.

Conventional meta-analytic models			WLS PET model		WLS PEESE model	
Fixed effects	Random effects	Q/*I*^2^ (heterogeneity)	Intercept	Slope	Intercept	Slope
0.24 (0.11, 0.37)	0.24 (0.11, 0.38)	24.69/6.8%	0.11 (-0.56, 0.78)	0.42 (-1.64, 2.48)	0.17 (-0.16, 0.49)	0.67 (-2.08, 3.42)

*WLS, weighted least squares; PET, precision-effects test; PEESE, precision effect estimate with SE. Numbers in parentheses are 95% confidence intervals. Random effects tests of heterogeneity, *p* > 0.05.*

**FIGURE 2:**
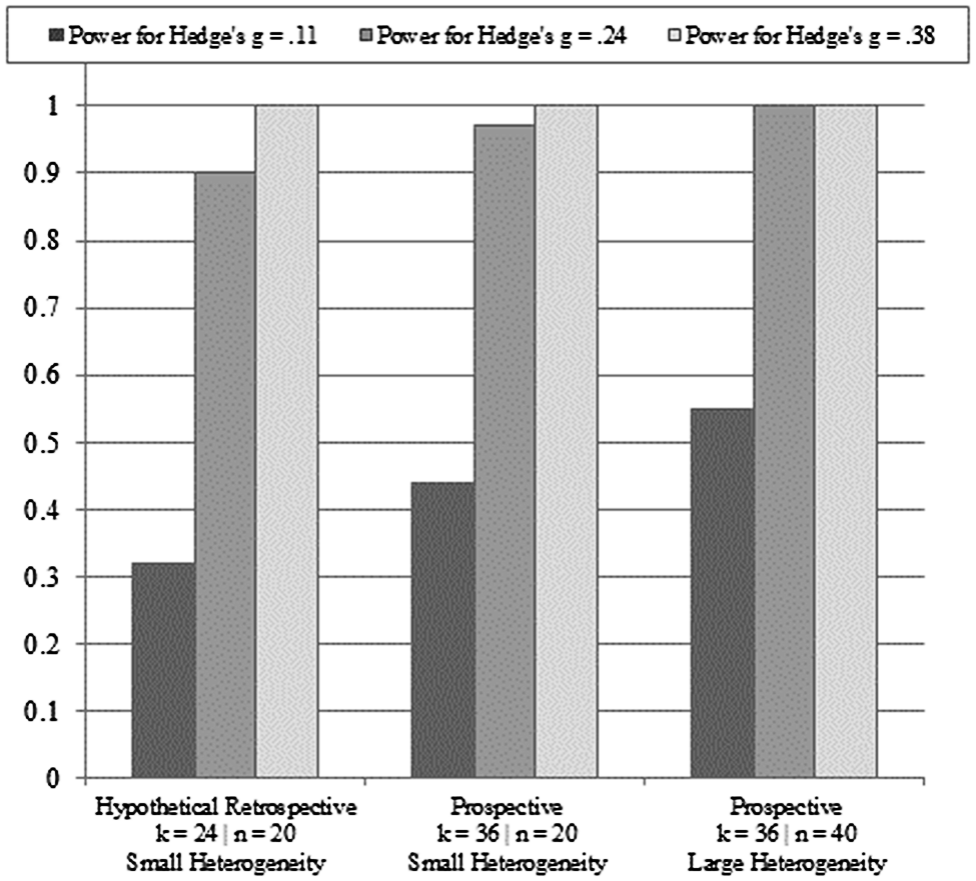
**Meta-analytic power analyses under varying assumptions for effect sizes, group sizes, heterogeneity, and number of studies**.

Based on the *post hoc* power analyses for the reported effect size (Hedge’s *g* = 0.24), the observed power for the mean reported group sample sizes was very low (1 - β = 0.11). Even under the hypothetical conditions of the upper ends of the ranges of group sizes serving as the mean group sizes, power would still be quite low (1 - β = 0.18). **Figure 2** displays the results of the retrospective and prospective meta-analytic power analyses under varying assumptions for effect sizes, group sizes, heterogeneity, and number of studies. For Hedge’s *g* = 0.11, the results showed that even when adding 12 more studies (i.e., *k* = 36), retaining small group sizes still resulted in an underpowered meta-analytic design (1 - β = 0.44). Doubling the group sizes and employing large heterogeneity improved the meta-analytic power estimate to 55% for Hedge’s *g* = 0.11, with *k* = 36. The retrospective and prospective meta-analytic power analyses for Hedge’s *g* = 0.24 showed approximately double the power of those observed for Hedge’s *g* = 0.11, with a similar pattern of stepwise increases in power as group size and number of studies increased. For Hedge’s *g* = 0.38, perfect power estimates were observed under all conditions (i.e., 1 - β = 1).

As an adjunct to the *post hoc* power analyses, PPV analyses were first conducted using the two average power estimates for the primary studies. The initial analysis used the mean *post hoc* power (1 - β = 0.11), α = 0.05, a very liberal (and unrealistic) R criterion (pre-study odds) of one in two tested effects being truly non-null [where *R* = 1/(2 - 1) = 1], and *n* = 24 (i.e., the number of samples with similar power addressing the same research question). Under these conditions, the PPV = 1(1- 0.89^24^)/(1 + 1 -[1 - 0.05]^24^ - (1)0.89^24^) = 0.57; meaning that among 24 studies with identical power, the probability that at least one discovered study effect is true is slightly greater than 50%. Using the hypothetical upper bound of power (1 - β = 0.18), the PPV increases to 0.58. Under a somewhat less liberal reading of pre-study odds of one in four [where *R* = 1/(4 - 1) = 0.33], the PPV for the mean *post hoc* power (1 - β = 0.11) was 0.30; meaning that among 24 studies with identical power, the probability that at least one discovered study effect is true is 30%. The PPV increased slightly (PPV = 0.32) under the hypothetical condition of the high end of *post hoc* power (1 - β = 0.18). Under a more conservative reading of pre-study odds of one in eight [where *R* = 1/(8 - 1) = 0.14], the PPV for the mean *post hoc* power (1 - β = 0.11) was 0.15; meaning that among 24 studies with identical power, the probability that at least one discovered study effect is true is 15%. Again, the PPV increased slightly (PPV = 0.17) under the hypothetical condition of the high end of *post hoc* power (1 - β = 0.18).

**FIGURE 3:**
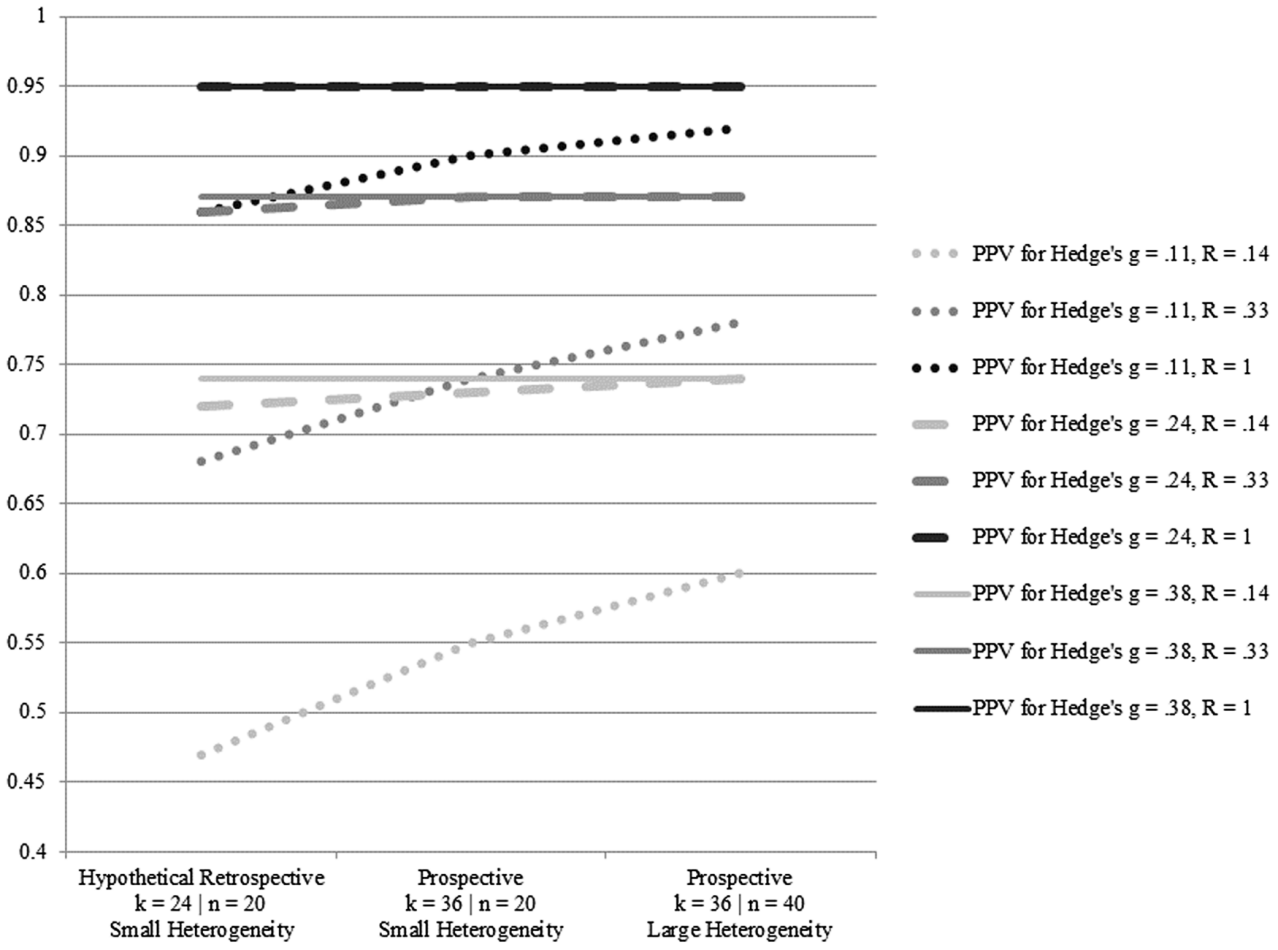
**Meta-analytic positive predictive value (PPV) analyses under varying assumptions for effect sizes, pre-study odds, group sizes, heterogeneity, and number of studies**.

**Figure 3** displays the meta-analytic PPV estimates under varying assumptions for effect sizes, pre-study odds, group sizes, heterogeneity, and number of studies. For Hedge’s *g* = 0.11 and conservative pre-study odds (one in eight), prospective meta-analytic PPV values were 0.55 and 0.60 across small versus large amounts of heterogeneity and group sample sizes of 20 versus 40. For Hedge’s *g* = 0.24 and conservative pre-study odds (one in eight), prospective meta-analytic PPV values (where *k* = 36) were both 0.74 across small versus large amounts of heterogeneity and group sample sizes of 20 versus 40. For Hedge’s *g* = 0.38 and conservative pre-study odds (one in eight), prospective meta-analytic PPV values (where *k* = 36) were both 0.95 across small versus large amounts of heterogeneity and group sample sizes of 20 versus 40. Increases in study odds produced prospective meta-analytic PPV values ranging from 0.74 to 0.95 across the three effect size estimates.

Finally, **Table 1** also displays the results of the fixed and random-effects models, as well as the intercept terms for the PET and the PEESE. The re-analysis of the random effects model using *R* produced the same estimate as that reported by Au et al. (2014; Hedges *g* = 0.24, *p* < 0.05). Both the PET and PEESE analyses produced effect size estimates that were smaller than the random effects estimate and whose confidence intervals included zero. Adhering to the conditional approach for interpreting and selecting PET or PEESE effects suggested by Stanley and Doucouliagos (2013), the PET estimate would be retained for analytic interpretation purposes due to it being statistically indistinguishable from zero. Below, we discuss why the PET-PEESE effects derived from the current base of evidence should be considered tentative, at best, and how these estimates would likely fluctuate as the literature expands.

## DISCUSSION

The goal of the present review was to evaluate and re-analyze the experimental evidence base for working memory training transfer effects to fluid intelligence originally identified and meta-analyzed by Au et al. (2014). Several analytic approaches were used to examine small-study effects related to publication bias in the presence of low power and small-study effects related to low power in the absence of publication bias. To summarize, there was no evidence found for small-study effects related to publication bias. However, there was evidence for small-study effects related to low power. The implications of these results for understanding possible transfer effects and meta-analytic planning, more generally, are discussed below.

The results of the *post hoc* power analyses indicated the reported studies suffer from a uniformly high rate of Type II errors, which is intrinsically tied to small-study effects related to low power. In broad terms, an observed power estimate of 11% suggests that in a research domain such as working memory training transfer effects, if 50 legitimately non-null effects could be detected, then approximately five effects, at this level of power, will actually be detected. Even under the hypothetical conditions of the uppers ends of the ranges for the groups, power was still far below the conventional benchmark for adequately powered studies (i.e., 1 - β = 0.80). The prospective meta-analytic power analyses produced considerably larger estimates under a variety of assumptions, but still produced estimates below 1 - β = 0.80 for Hedge’s *g* = 0.11, especially under the assumption that group sizes remain at *n* = 20 for an additional 12 studies (i.e., *k* = 36). Taken together, the *post hoc* power analyses of the primary studies and the meta-analytic power estimates are suggestive of (1) a highly underpowered current base of evidence, and (2) a base of evidence that, while likely to experience growth in the coming years, might disrupt future meta-analytic efforts if the true effect size is smaller than that observed by Au et al. (2014) and sample sizes remain the same (on average). The effect of low power in small studies extends beyond the ability to avoid erroneously rejecting a truly non-null effect.

For those underpowered studies that do detect an effect, such results can be characterized as a “winner’s curse” (Ioannidis, 2008). The results of the PPV analyses provide some support for this contention, showing that even under very liberal (and unrealistic) assumptions about pre-study odds of one in two, the very low observed power in the studies identified by Au et al. (2014) indicated that among 24 studies of working memory training, the probability of a single study among them showing a true effect was slightly more than 50%. Given the exploratory nature of the working memory training transfer effect paradigm, a more conservative rendering of pre-study odds of one in eight resulted in a reduction of the PPV estimate to a 15% probability that a single study among 24 studies would show a true effect. As described above, the PPV analysis used here does not correct for potential sources of bias (i.e., in this sense, it is a more liberal estimate). However, it should be noted that, by design, the computation for this PPV analysis includes a compounding correction (by number of studies) for Type I error rates that produces a ceiling for PPV estimates. This means that as the number of studies increases, increases in power alone will not offset the multiple-tests correction for Type I errors and, as a consequence, PPV estimates will form an asymptotic function (cf. Ioannidis, 2005). A complementary set of retrospective and prospective meta-analytic PPV analyses produced larger estimates – especially for the conditions associated with Hedge’s *g* = 0.24 and 0.38, where PPV values topped out at 0.95 under multiple conditions. These PPV results suggest there should be little concern going forward as to the robustness of future meta-analytic efforts if the true effect is Hedge’s *g* = 0.24 or larger. However, the prospective meta-analytic PPV estimates were considerably lower for Hedge’s *g* = 0.11, under conditions of low pre-study odds and the continued use of *n* = 20 for treatment and control group sizes. The latter meta-analytic PPV estimates suggest future meta-analytic efforts might be undermined if the true effect is closer to the lower bound of the current observed confidence interval identified by Au et al. (2014). Taken together, these two sets of PPV analyses – when tested using conservative assumptions – are suggestive of small-study effects related to low power in the absence of publication bias.

Under conditions of very low power, the only effects that can be detected are large to very large effects; precisely the types of effects that are unlikely to reflect true results for many research questions. This is considered a “curse” because the detected effect is, at the very least, artifactually inflated, and, at worst, entirely spurious. Moreover, the winner’s curse can propagate a misguided search for replication, where the continued use of small studies to reproduce or expand on the original effect is undermined by the improper anchoring of the original effect size estimate as the criterion for replication. Relatedly, and as is evidenced by the range of effect sizes located by Au et al. (2014; Hedge’s *g* range, -0.28 to 1.10), small studies are more likely to show a vibration of effects (Ioannidis, 2008), where inconsistencies in analytic approaches and tests (removing versus retaining outliers, controlling for covariates), as well as measurement variation (e.g., varying assessments of fluid intelligence), can have an undue effect on the range of observed estimates compared to studies that use larger samples. It should be noted though, that even with few conventionally significant effects, very wide confidence intervals, and some vibration of the observed effects, there was still visual evidence of small positive skew among the individual study effects depicted in the forest plot presented by Au et al. (2014). In other words, the approximate center of the vibration of effects was not zero (or negative).

The results of the PET-PEESE analyses provided a tentative quantification of the impact of small-study effects by producing an estimate of the working memory training transfer effect that was smaller than the effect that was observed by Au et al. (2014) and was not different from zero. Recent work suggests these regression-based approaches provide the least biased estimate of an underlying effect (e.g., Stanley, 2008; Moreno et al., 2009; Rücker et al., 2011; Stanley and Doucouliagos, 2013; Carter and McCullough, 2014). However, it should be emphasized that the extent of the downward correction suggested by the PET-PEESE intercepts (and the wide extent of the respective confidence intervals) is computationally informed by the lack of an association between the SE and effect sizes (i.e., the non-significant slope terms describe above). This lack of an association between SE and effect sizes is, in turn, related to the limited variability among the SE (as indicated by the very low levels of heterogeneity across the studies and seen in the horizontal banding of the SE depicted in **Figure 1**). The lack of significant variability in SE is, in turn, computationally linked to the uniformly small sample sizes of the studies identified by Au et al. (2014). Given the present base of evidence, the implication of these linkages is that the PET-PEESE estimates are likely unstable. As the working memory training literature expands, PET-PEESE estimates will likely show fluctuations in response to greater variability in SE (ideally tied to the employment of larger sample sizes).

Similarly, the random-effects model estimate and its confidence interval might also show fluctuations. It is noteworthy that the meta-analytic effect size estimates were identical across the fixed-effects and random-effects models. Moreover, the confidence intervals were nearly identical, indicating the weighting of the random-effects model was entirely unaffected by the use of the between-studies source of sampling error owing to the uniformly large amounts of within-studies sampling error across the studies (i.e., the imprecision of the studies accounted for all of the between-studies variability). Computationally, this resulted in highly similar weighting across both the fixed-effects and random-effects models (which is also easily inferred by calculating the unweighted simple mean, Hedge’s *g* = 0.258, which only differs slightly from the weighted estimates). Au et al. (2014) attributed the lack of heterogeneity in the results to the intervention and sample parameters (i.e., *n*-back training interventions deployed among healthy young adults). The use of varying *n*-back training tasks (e.g., single versus dual), measures of fluid intelligence, etc., would seem at odds with such an explanation. Computationally, the unusually low levels of heterogeneity are primarily related to the imprecision of the collected studies, which, in turn, is tied to the uniformly low power across the studies. The inclusion of greater powered studies in the meta-analytic database would necessarily improve heterogeneity and result in a widening of the confidence interval for the random-effects model as compared to the confidence interval for the fixed-effects model. As it is, the imprecision in the collected studies had the ironic effect of reducing between-studies variance to nil and rendering the random-effects model a fixed-effects model, which, as suggested above, runs counter to the apparent diversity of researchers, implementations, measures, etc., used to examine the working memory training paradigm. That being stated, the random-effects estimated identified by Au et al. (2014) and reported herein does provide the most precise estimate of the working memory training transfer effect to date. However, it is also clear this estimate is derived from a uniformly low-powered set of primary studies.

To a certain extent, it is challenging to offer more than computation and coarse speculation regarding the sources of these small-sample studies. One of the possible barriers to employing adequately powered designs is the rapid formation of research encampments following a novel, exploratory, provocative, and/or preliminary research finding (cf. Ioannidis and Trikalinos, 2005). While effect validation across researchers and laboratories is essential to reliable science, the process of cross-validation can be conducted using a model of rapid deployment and dissemination, which might include faithful attempts at reproducing materials, methods, and procedures, but (following the lead of the initial study) also grossly underpowered designs. There is some indication of this in the studies identified by Au et al. (2014). The data for the 24 effects were collected in a short span (approximately 6 years) by more than 10 research groups. The vast majority of the studies appeared in peer reviewed publications; meaning the conduct of the studies (including the acquisition of materials, human subjects approval, staff training, recruitment, assessment, and training), data entry and analyses, as well as manuscript preparation, submission, and revision all occurred during a constrained timeframe (especially considering some researchers produced multiple published studies of the training effect during this time). Assuming improper anchoring of the initial effect size, most attempts at reproducibility would continue to be substantially underpowered. Whether it was due to anchoring, a model of rapid deployment and dissemination, and/or impassivity regarding considerations of statistical power, all of the studies reported by Au et al. (2014) used underpowered designs. As others have examined elsewhere, the early returns of meta-analytic efforts are often inflated or lack credibility (Pereira and Ioannidis, 2011). It is often not until a literature has matured that more credible meta-analytic estimates can be produced.

As it specifically relates to the approach of Au et al. (2014), it should be noted that additional moderator and meta-regression analyses were reported, despite the absence of evidence for statistical heterogeneity. As can be seen in **Table 1** and as was reported by Au et al. (2014), the test for heterogeneity was non-significant and did not reach conventional guidelines for low amounts of statistical heterogeneity (i.e., *I*^2^ ~ 25%; Higgins et al., 2003). Nevertheless, given the various implementations of the training paradigm used in the primary studies, Au et al. (2014) conducted these analyses to examine possible differences in transfer effects. A potential outcome of these analyses and related reporting is the dissemination of the additional effects as both reliable and interpretable. Given the findings of the present work, the additional moderator and meta-regression results reported by Au et al. (2014) should be interpreted with due caution and be considered especially provisional.

The potential benefit of the approaches reported herein is not only critical evaluation, but also constructive guidance. One of the contributions of the work of Au et al. (2014) is the collection of studies in an emerging and contentious literature. As appears clear now, this literature was at a somewhat limited stage of adequacy in design methodology and measurement coherence to allow for the application of meta-analytic methods. What is particularly clear is that the primary studies were only adequately powered to detect large or very large effects. Furthermore, comparisons across these studies were somewhat hindered by the diversity of measures used to assess fluid intelligence (although given the imprecision of the studies, these seemingly non-trivial differences did not contribute to statistical heterogeneity). **Figure 2** displays scenarios related to low power as the working memory training literature accrues a larger base of primary studies. Specifically, if the true effect is smaller than that estimated by Au et al. (2014), then future meta-analytic efforts are likely to be underpowered, even with a twofold increase in average group sizes and a 50% increase in studies. Conversely, if the true effect is only slightly smaller (or larger, of course) than that estimated by Au et al. (2014), then future meta-analytic work will be more than sufficiently powered. As a matter of conservative research practice, it seems advisable to adopt greater-powered designs for primary studies of the working memory training transfer effect in the case that the smaller estimate proves closer to the center of the underlying distribution of effects (assuming a random-effects model).

There is little question that the implementation of these training paradigms is time-consuming and costly. These logistical considerations should provide even further impetus to pursue high-powered designs that are directly comparable within and across research labs. This would require a good deal of internal and external coordination, but would likely short-circuit the ‘volleying’ pattern of studies seen in many areas of research contention. Only when well-powered studies using commensurate designs, measures, and analyses are conducted will it be possible to more precisely estimate and evaluate working memory training as a viable mechanism for improving fluid intelligence. Moreover, high-powered designs will not only be required to evaluate the robustness of the general transfer effect, but are especially critical for more nuanced explorations of individual difference and other potentially moderating factors of the transfer effect (cf. Jaeggi et al., 2014).

Given the perceived definitive nature of meta-analyses, mistimed, or misapplied meta-analytic techniques are a serious concern for all scientific disciplines. We close this review with a list of literature features and statistical planning components that warrant consideration while planning a meta-analysis. Aside from the important meta-analytic considerations outlined by others (cf. Schmidt, 2013), we recommend seven supplementary meta-analytic planning tasks in **Table 2**. Attending to these considerations should aid researchers in making a determination as to whether conducting a meta-analysis (versus a critical narrative review) is constructive or whether the (in)adequacy of the literature would lead to unreliable or biased estimates that would further obfuscate the existence or magnitude of an effect of interest.

**Table 2:** Recommendations for supplementary meta-analytic planning metrics.

Additional tasks for evaluating literature suitability for meta-analytic treatment
✓ Calculate *post hoc* power estimates for the primary studies.
✓ Conduct an *a priori* meta-analytic power analysis ( Valentine et al., 2010; Hedges and Pigott, 2001).
✓ Assess the prevalence of direct replication attempts in the literature.
✓ Assess the prevalence of null findings in the published literature. ✓ Examine the discrepancy in the ratio of null-to-significant findings using binomial tests (Ioannidis and Trikalinos, 2007).
✓ Examine and quantify design and measurement quality and commensurability across studies (via expert coding rubric).
✓ Survey the prevalence of study and/or data registration of the primary studies, especially for randomized trials and interventions.

## Conflict of Interest Statement

The authors declare that the research was conducted in the absence of any commercial or financial relationships that could be construed as a potential conflict of interest.

## SUPPLEMENTARY MATERIAL

The Supplementary Material for this article can be found online at:
