# Supplementary material for: Reliable gains? Evidence for substantially underpowered designs in studies of working memory training transfer to fluid intelligence
Source: Front Psychol. 2015 Jan 22;5:1589. doi: 10.3389/fpsyg.2014.01589 (PMC4302828; doi:10.3389/fpsyg.2014.01589)
Supplement: Supplementary file 1 [file Data_Sheet_1.DOCX]

**Annotated R scripts for analyses**

# The following packages should be loaded: Formula, Matrix, meta, metafor, pwr #

# Hedge’s gs, SEs, and variances (SE-squared) as reported in Figure 3 of Au et al. (doi:10.3758/s13423-014-0699-x); k = 24. #

g<-c(.023, -.208, .645, .212, .389, .552, -.019, .640, .283, 1.109, .220, .184, .054, .337, -.280, -.053, .759, .816, .263, .349, -.070, -.157, .416, -.276)

g.se<-c(.413, .399, .335, .265, .359, .261, .300, .486, .412, .512, .489, .274, .253, .556, .363, .273, .276, .332, .298, .308, .264, .440, .275, .316)

g.v<-c(.170569, .159201, .112225, .070225, .128881, .068121, .09, .236196, .169744, .262144, .239121, .075076, .064009, .309136, .131769, .074529, .076176, .110224, .088804, .094864, .069696, .1936, .075625, .099856)

# Mean treatment (n = 19.96) and control (n = 19.29) group subsample sizes, as reported by Au et al.; means used in post hoc power analyses below. #

n1.all<-c(19.96, 19.96, 19.96, 19.96, 19.96, 19.96, 19.96, 19.96, 19.96, 19.96, 19.96, 19.96, 19.96, 19.96, 19.96, 19.96, 19.96, 19.96, 19.96, 19.96, 19.96, 19.96, 19.96, 19.96)

n2.all<-c(19.29, 19.29, 19.29, 19.29, 19.29, 19.29, 19.29, 19.29, 19.29, 19.29, 19.29, 19.29, 19.29, 19.29, 19.29, 19.29, 19.29, 19.29, 19.29, 19.29, 19.29, 19.29, 19.29, 19.29)

# Upper ends of treatment (n = 36) and control (n = 43) group subsample sizes, as reported by Au et al., used in hypothetical power estimate of effect at upper range of reported group sizes

n1a.all<-c(36, 36, 36, 36, 36, 36, 36, 36, 36, 36, 36, 36, 36, 36, 36, 36, 36, 36, 36, 36, 36, 36, 36, 36)

n2a.all<-c(43, 43, 43, 43, 43, 43, 43, 43, 43, 43, 43, 43, 43, 43, 43, 43, 43, 43, 43, 43, 43, 43, 43, 43)

# Conventional fixed and Random-effects models #

meta.all<-metagen(g, g.se)

# Fixed and Random effects results #

meta.all

# Funnel Plot #

png(file="funnel.png", width = 1600, height = 1400, res = 200)

layout(matrix(c(1,1,2,3,4,5), 3, 2, byrow = TRUE), respect = TRUE)

funnel(meta.all, lty.fixed = 1, lty.random = 5, xlim = c(-1.5, 1.5), xlab = "Effect size (Hedge's g)", ylab = "Standard Error (SE)", cex = .75, col = 1, bg = 1, contour=c(0.00001, 0.95), col.contour=c("grey", "white"), family = "serif", cex.lab = 1.5, main = "k = 24", cex.main = 1.8)

# Calculating average power based on post-hoc power calculations, assuming RE estimate of g = .24 for full sample (k = 24) #

P.24.all<-mapply(pwr.t2n.test, d=.24, n1 = n1.all, n2 = n2.all)

PowP.24.all<-sapply(P.24.all[5,1:24], as.numeric)

MeanPower.24.all<-mean(PowP.24.all)

MeanPower.24.all

# Deriving individual CIs for each experiment and the number of studies where p > .05 #

ci.all<-data.frame(ci(TE = g, seTE = g.se))

ci.all

NumNonSig.all<-with(ci.all, c(sum(p>.05)))

NumSig.all<-24-NumNonSig.all

NumSig.all

# p value for binomial test #

Powers.all<-c(MeanPower.24.all)

Powers.all

BinomPow.all<-mapply(binom.test, NumSig.all, 24, Powers.all, conf.level = .9, alternative = "greater")

p.values.all<-sapply(BinomPow.all[3,1:1], as.numeric)

p.values.all

# Calculating average power based on post-hoc power calculations, using upper end of group ranges, assuming RE estimate of g = .24 for full sample #

P.24.all<-mapply(pwr.t2n.test, d=.24, n1 = n1a.all, n2 = n2a.all)

PowP.24.all<-sapply(P.24.all[5,1:24], as.numeric)

MeanPower.24.all<-mean(PowP.24.all)

MeanPower.24.all

# Meta-analytic power estimate based on Hedge's g and SE #

maPow = function(g,se,alpha){

c=qnorm(1-alpha/2) #assumes a two-tailed test

lambda=(g-0)/se

return(1-pnorm(c-lambda)+pnorm(-c-lambda))

}

# PPV for a meta-analysis #

maPPV=function(g,se,R,alpha){

P=maPow(g,se,alpha)

beta=1-P

return(((1-beta)*R)/((1-beta)*R + alpha))

}

# Meta-analytic power and PPV for Hedge's g = .11, k = 24, n1,n2 = 20, small heterogeneity (lambda calculated using convention of tau-squared = .33) #

maPow(0.11,.074,.05) # Power #

maPPV(0.11,.074,.14,.05) # PPV with R = .14 #

maPPV(0.11,.074,.33,.05) # PPV with R = .33 #

maPPV(0.11,.074,1,.05) # PPV with R = 1 #

# Meta-analytic power and PPV for Hedge's g = .11, k = 36, n1,n2 = 20, small heterogeneity (lambda calculated using convention of tau-squared = .33) #

maPow(0.11,.061,.05) # Power #

maPPV(0.11,.061,.14,.05) # PPV with R = .14 #

maPPV(0.11,.061,.33,.05) # PPV with R = .33 #

maPPV(0.11,.061,1,.05) # PPV with R = 1 #

# Meta-analytic power and PPV for Hedge's g = .11, k = 36, n1,n2 = 40, large heterogeneity (lambda calculated using convention of tau-squared = 1) #

maPow(0.11,.053,.05) # Power #

maPPV(0.11,.053,.14,.05) # PPV with R = .14 #

maPPV(0.11,.053,.33,.05) # PPV with R = .33 #

maPPV(0.11,.053,1,.05) # PPV with R = 1 #

# Meta-analytic power and PPV for Hedge's g = .24, k = 24, n1,n2 = 20, small heterogeneity (lambda calculated using convention of tau-squared = .33) #

maPow(0.24,.074,.05) # Power #

maPPV(0.24,.074,.14,.05) # PPV with R = .14 #

maPPV(0.24,.074,.33,.05) # PPV with R = .33 #

maPPV(0.24,.074,1,.05) # PPV with R = 1 #

# Meta-analytic power and PPV for Hedge's g = .24, k = 36, n1,n2 = 20, small heterogeneity (lambda calculated using convention of tau-squared = .33) #

maPow(0.24,.061,.05) # Power #

maPPV(0.24,.061,.14,.05) # PPV with R = .14 #

maPPV(0.24,.061,.33,.05) # PPV with R = .33 #

maPPV(0.24,.061,1,.05) # PPV with R = 1 #

# Meta-analytic power and PPV for Hedge's g = .24, k = 36, n1,n2 = 40, large heterogeneity (lambda calculated using convention of tau-squared = 1) #

maPow(0.24,.052,.05) # Power #

maPPV(0.24,.052,.14,.05) # PPV with R = .14 #

maPPV(0.24,.052,.33,.05) # PPV with R = .33 #

maPPV(0.24,.052,1,.05) # PPV with R = 1 #

# Meta-analytic power and PPV for Hedge's g = .38, k = 24, n1,n2 = 20, small heterogeneity (lambda calculated using convention of tau-squared = .33) #

maPow(0.38,.074,.05) # Power #

maPPV(0.38,.074,.14,.05) # PPV with R = .14 #

maPPV(0.38,.074,.33,.05) # PPV with R = .33 #

maPPV(0.38,.074,1,.05) # PPV with R = 1 #

# Meta-analytic power and PPV for Hedge's g = .38, k = 36, n1,n2 = 20, small heterogeneity (lambda calculated using convention of tau-squared = .33) #

maPow(0.38,.061,.05) # Power #

maPPV(0.38,.061,.14,.05) # PPV with R = .14 #

maPPV(0.38,.061,.33,.05) # PPV with R = .33 #

maPPV(0.38,.061,1,.05) # PPV with R = 1 #

# Meta-analytic power and PPV for Hedge's g = .38, k = 36, n1,n2 = 40, large heterogeneity (lambda calculated using convention of tau-squared = 1) #

maPow(0.38,.052,.05) # Power #

maPPV(0.38,.052,.14,.05) # PPV with R = .14 #

maPPV(0.38,.052,.33,.05) # PPV with R = .33 #

maPPV(0.38,.052,1,.05) # PPV with R = 1 #

# Precision-effect test (PET) #

SE.all<-lm(g~g.se, weights = 1/g.v)

summary(SE.all)

confint(SE.all)

# Precision effect estimate with standard error (PEESE) #

V.all<-lm(g~g.v, weights = 1/g.v)

summary(V.all)

confint(V.all)
